# Portal Hypertension in Children: Investigating Umbilical Catheterization in the Neonatal Period

**DOI:** 10.7759/cureus.66060

**Published:** 2024-08-03

**Authors:** Nadia Bouhafs, Amal Hamami, Aziza Elouali, Abdeladim Babakhouya, Maria Rkain

**Affiliations:** 1 Department of Pediatrics, Faculty of Medicine and Pharmacy of Oujda, Mohammed VI University Hospital Oujda, Oujda, MAR; 2 Pediatric Gastroenterology, Centre Hospitalier Universitaire Mohammed VI Oujda, Oujda, MAR

**Keywords:** umbilical catheterization, portal cavernoma in children, portal vein thrombosis, esophageal varices, hematemesis

## Abstract

Portal cavernoma is a major cause of extrahepatic portal hypertension (EHPH) in children. It is a serious condition, due to the frequency and severity of digestive hemorrhages secondary to the rupture of esophageal varices (EV). Neonatal umbilical catheterization is a significant risk factor for the development of portal vein thrombosis (PVT) and portal hypertension. We report a case of a five-year-old male who presented with upper gastrointestinal (GI) bleeding on ruptured esophageal varices resulting from a portal cavernoma, complicating neonatal umbilical vein catheterization. This case illustrates the risk of severe vascular complications, particularly portal hypertension that can result from neonatal umbilical vein catheterization.

## Introduction

Portal cavernoma (synonymous with portal vein thrombosis, PVT) comprises a network of veins whose caliber, initially millimetric or microscopic, is increased, thereby facilitating the flow of hepatopetal portal blood. This condition results from a natural compensatory mechanism that is triggered when the portal vein becomes obstructed. It is the consequence of thrombotic and chronic occlusion of the extrahepatic portal system [1]. Although the exact causes of PVT in children remain unknown, several risk factors have been identified and classified into three main groups: general causes (procoagulant state), local factors (abdominal infection, omphalitis, umbilical catheter), and vascular malformations [2]. Umbilical venous catheterization itself represents a risk factor for the development of PVT. In this report, we discuss a case of portal thrombosis in a five-year-old male due to umbilical catheterization, which was revealed at a later age. By sharing this clinical experience, we aim to raise awareness among healthcare professionals about the risks associated with umbilical catheterization and to promote optimized clinical practices to minimize these risks.

## Case presentation

A five-year-old male presented to the pediatric emergency department two days after an episode of hematemesis, during which he had vomited an estimated 500 ml of blood. He had been born at a gestational age of 39 weeks with a birth weight of 3000 g following an uncomplicated pregnancy. His medical history revealed no abnormalities other than recurrent abdominal pain. The patient appeared pale on physical examination with discolored conjunctivae and tachycardia at 122 bpm; the other vital signs were normal. He had a palpable splenomegaly 2 cm below the rib cage, without hepatomegaly. There were no signs of chronic liver disease. The initial blood work showed severe anemia with thrombocytopenia (hemoglobin: 5.4 g/dl, normochromic normocytic, platelets: 118,000/µl, normal blood smear) and a prothrombin level of 62%. Liver function tests and coagulation profiles were normal (Table 1).

**Table 1:** Results of biological tests at admission ALT: alanine aminotransferase; AST: aspartate aminotransferase; GGT: gamma-glutamyl transferase; MCHC: mean corpuscular hemoglobin concentration; MCV: mean corpuscular volume

Parameters	Results	References range
Hemoglobin (g/dl)	5.4	9.5-13.5
MCV (fL)	83	80-100
MCHC (g/dL)	32	32-36
White blood cell count (E/mm^3^)	3170	5000-10,000
Platelets (E/mm^3^)	118,000	150,000-400,000
Prothrombin level (%)	62	70-120
AST (IU/L)	29	8-30
ALT (IU/L)	18	8-35
GGT (IU/l)	11	10-71

**Figure 1:**
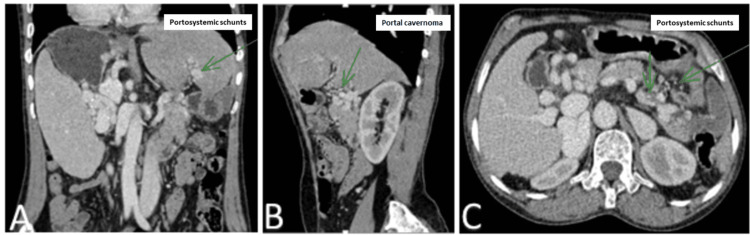
CT angiography images in axial (A), parasagittal (B), and coronal (C) sections showing a portal cavernoma with portosystemic shunts CT: computed tomography

An esophagogastroduodenoscopy revealed multiple stage 3 esophageal and fundic varices, with no active bleeding. CT angiography showed a portal cavernoma with signs of portal hypertension (Figure 1).

**Figure 2:**
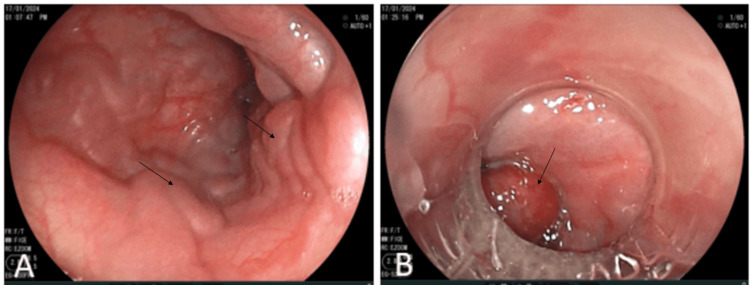
Endoscopic images showing esophageal varices in the lower third of the esophagus before (A) and after (B) endoscopic ligation

Initially, the origin of the portal cavernoma was unclear. The patient's history did not indicate a predisposition to thrombosis; the thrombophilia assessment showed normal results and no vascular malformations. However, neonatal records revealed an admission to the neonatal ICU for neonatal asphyxia due to maternal-fetal infection and unconjugated hyperbilirubinemia due to rhesus incompatibility, complicated by nosocomial septicemia for which he had spent 28 days in the neonatal. An umbilical catheter had been inserted for transfusion and treatment for over 10 days. No complications had been reported in the following years. Our patient was treated with Sandostatin and beta-blockers and underwent three sessions of esophageal variceal ligation (Figure 2) with favorable initial progress while awaiting surgery.

## Discussion

Portal cavernoma was first described in 1903 during the autopsy of a 44-year-old patient who died due to extensive mesenteric venous thrombosis [3]. In children, portal cavernoma is a major cause of "pre-hepatic" portal hypertension. It is a grave condition given the high rate and severity of digestive bleeding due to the rupture of esophageal varices (EV). In the United States, its incidence is estimated at 1% in the general population while a study in Morocco reported an incidence of about 1.45% [1]. Umbilical vein catheterization is a major risk factor for the development of portal cavernoma in children, with a rising prevalence (~60-70%) due to the procedure's widespread use in neonatal care [2]. A systematic review of the literature has revealed the considerable risk of PVT related to umbilical catheterization [4]. The combined average rate of neonatal PVT due to umbilical vein catheters (UVC) across studies was found to be 12% (range: 0-49%). However, a more recent multicenter survey including 187 children diagnosed with PVT (average age at diagnosis: four years) reported a history of neonatal UVC placement in 65% of cases [5].

Scientific literature highlights that UVC-related PVT is mainly associated with improper catheter tip placement [4]. The UVC should be centrally positioned, ideally at the junction between the inferior vena cava and the right atrium. Indeed, if the catheter tip is too low, it could be responsible for necrotizing enterocolitis, colon perforation, liver abscess, and PVT [6]. Therefore, careful evaluation of the UVC tip position is necessary to reduce catheter-related complications. A recent study found that an ultrasound assessment of UVC's position helped identify catheter position in 100% of cases compared with radiographic assessment [7]. Prospective studies have concluded that properly inserted UVCs do not cause PVT [4].

Besides improper catheter placement, which is the primary risk factor, several other factors can increase the risk of PVT in catheterized newborns. These factors include sepsis, trauma during insertion, transfusions, infusion of hypertonic solution [8-9], and solutions with significantly elevated serum calcium concentrations [10]. UVC-related PVT can sometimes resolve spontaneously. Kim et al.'s study involving 100 newborns found that 43% had asymptomatic PVT, with complete resolution in 56% during follow-up. The majority of cases remain unrecognized and are discovered later between the ages of 6-10 years, as in our patient [8].

Clinical signs of portal cavernoma are related to extrahepatic portal hypertension (EHPH). Gastrointestinal (GI) hemorrhages and splenomegaly are the most common clinical manifestations. Around 50% of children initially present with GI bleeding. Splenomegaly is found in over 90% of children with PVT, and ascites may be present in 8.3% of cases [11,12]. Growth retardation exists in 50% of the cases. The decreased hepatic portal flow and/or resistance to growth hormone have been proposed as pathophysiological hypotheses to explain this scenario [13]. Our patient had normal weight and height. Biologically, splenomegaly is accompanied by hypersplenism [11]. Chronic anemia is found in almost all cases, and thrombocytopenia and decreased prothrombin time (PT) are secondary to overconsumption by a mechanism of disseminated intravascular coagulation (DIC) within the capillaries forming the cavernoma. Liver function tests are generally unchanged. Abdominal Doppler ultrasound is the most commonly used diagnostic test, with a sensitivity of over 90%. Upper GI endoscopy must be performed in all cases, as it can reveal the presence of esophagogastric varices in approximately 84% of cases [11,14].

None of the studies in the literature encourage the use of beta-blockers in children to prevent digestive hemorrhage [15]. On the other hand, the effectiveness of primary prophylactic endoscopic treatment has been demonstrated [16]. Porto-systemic shunting is indicated if hemorrhagic complications persist despite endoscopic treatment, or in cases of symptomatic cholangiopathy. Mesenterico-Rex shunting or portal reperfusion, a more physiological technique, has shown promising results in the curative treatment of portal cavernomas [17].

Prevention of portal thrombosis related to umbilical catheterization in newborns is crucial. This involves correct catheter insertion, regular assessment of catheter position every 48 hours, close monitoring of newborns, especially those with additional risk factors, and minimizing the duration of catheter use (seven days maximum). If long-term central venous access (>4 days) is necessary, replacing the umbilical venous catheter with a percutaneous central venous catheter (PICC) or using ultrasound-guided central venous access may be beneficial. Manual liver retraction during the placement of an umbilical venous catheter improves the positioning rate of the UVC tip in the inferior vena cava. This maneuver is quick, easy to implement, and not associated with any adverse effects [18].

## Conclusions

This case report illustrates portal hypertension as a late complication of invasive neonatal procedures. PVT secondary to umbilical catheterization is a significant complication requiring sharp vigilance. Raising awareness among healthcare professionals about the risks associated with umbilical catheterization and closely monitoring children who have undergone this procedure is essential for optimal management and prevention of thrombotic complications.
